# Implementation of the WHO multimodal Hand Hygiene Improvement Strategy in a University Hospital in Central Ethiopia

**DOI:** 10.1186/s13756-016-0165-9

**Published:** 2017-01-05

**Authors:** Frieder Pfäfflin, Tafese Beyene Tufa, Million Getachew, Tsehaynesh Nigussie, Andreas Schönfeld, Dieter Häussinger, Torsten Feldt, Nicole Schmidt

**Affiliations:** 1 Department of Gastroenterology, Hepatology and Infectious Diseases (DGHID), Heinrich Heine University, Düsseldorf, Germany; 2 Hirsch Institute of Tropical Medicine, research and training centre of DGHID, operated in cooperation with Arsi University, Asella, Ethiopia; 3 Department of Infectious Diseases and Pulmonary Medicine, Charité – Universitätsmedizin Berlin, Augustenburger Platz 1, 13353 Berlin, Germany; 4 Arsi University, Asella, Ethiopia; 5 Institute of Tropical Medicine and International Health, Charité – Universitätsmedizin Berlin, Berlin, Germany; 6 Department for Infectious Disease Epidemiology, Robert Koch Institute, Berlin, Germany

**Keywords:** Hand hygiene, Ethiopia, World Health Organization, Infection control, Health-care worker, Alcohol-based handrub

## Abstract

**Background:**

The burden of health-care associated infections in low-income countries is high. Adequate hand hygiene is considered the most effective measure to reduce the transmission of nosocomial pathogens. We aimed to assess compliance with hand hygiene and perception and knowledge about hand hygiene before and after the implementation of a multimodal hand hygiene campaign designed by the World Health Organization.

**Methods:**

The study was carried out at Asella Teaching Hospital, a university hospital and referral centre for a population of about 3.5 million in Arsi Zone, Central Ethiopia. Compliance with hand hygiene during routine patient care was measured by direct observation before and starting from six weeks after the intervention, which consisted of a four day workshop accompanied by training sessions and the provision of locally produced alcohol-based handrub and posters emphasizing the importance of hand hygiene. A second follow up was conducted three months after handing over project responsibility to the Ethiopian partners. Health-care workers’ perception and knowledge about hand hygiene were assessed before and after the intervention.

**Results:**

At baseline, first, and second follow up we observed a total of 2888, 2865, and 2244 hand hygiene opportunities, respectively. Compliance with hand hygiene was 1.4% at baseline and increased to 11.7% and 13.1% in the first and second follow up, respectively (p < 0.001). The increase in compliance with hand hygiene was consistent across professional categories and all participating wards and was independently associated with the intervention (adjusted odds ratio, 9.18; 95% confidence interval 6.61-12.76; p < 0.001). After the training, locally produced alcohol-based handrub was used in 98.4% of all hand hygiene actions. The median hand hygiene knowledge score overall was 13 (interquartile range 11–15) at baseline and increased to 17 (15–18) after training (p < 0.001). Health-care workers’ perception surveys revealed high appreciation of the different strategy components.

**Conclusion:**

Promotion of hand hygiene is feasible and sustainable in a resource-constrained setting using a multimodal improvement strategy. However, absolute compliance remained low. Strong and long-term commitment by hospital management and health-care workers may be needed for further improvement.

**Electronic supplementary material:**

The online version of this article (doi:10.1186/s13756-016-0165-9) contains supplementary material, which is available to authorized users.

## Background

Hand hygiene is referred to as either hand washing with soap and water or hand disinfection. Important benefits of proper hand hygiene include reduction of nosocomial infections [1], reduced transmission of multi-drug resistant pathogens [2, 3], and cost effectiveness [4, 5]. Alcoholic handrub is regarded to be superior to washing hands with soap and water. It has greater activity against microorganisms, less time constraints, and better skin tolerability [5–7]. Furthermore, alcoholic handrub is better accessible in most settings as it can be provided in pocket bottles and may thus be available at any time at the point of care. The World Health Organization (WHO) has identified formulations for the local preparation of alcohol-based handrubs with substantially lower costs compared to commercial products [8].

Compliance with hand hygiene varies greatly between countries and settings but is globally low [5]. Several factors have been shown to be related to low compliance with hand hygiene in developed countries [9]. In low-income countries the major reason for non-compliance with hand hygiene may be lack of adequate facilities [10]. The burden of health-care associated infections (HAIs) is high in developing countries [11]. WHO has established a multimodal implementation strategy to improve compliance with hand hygiene [12]. Furthermore, the concept “my five moments for hand hygiene“was developed to perform hand hygiene in key moments [13]. Allegranzi et al. found that the implementation of WHO’s hand-hygiene strategy is feasible and sustainable in different settings and countries and leads to significant compliance and knowledge improvement in health-care workers (HCWs) [14]. There are, however, few data on the implementation of the WHO multimodal hand hygiene improvement strategy in Ethiopia, a country with high rates of nosocomial infections [15].

The main objective for this study was to assess compliance with hand hygiene in selected wards of the Asella Teaching Hospital (ATH) before and after the implementation of the hand hygiene campaign. The secondary objectives were to assess compliance with hand hygiene for the different professional categories and the different wards and to assess perception and knowledge for the different professional categories before and after the implementation of the hand hygiene campaign.

## Methods

### Study setting

The study was carried out in selected wards of ATH in the Arsi Zone, Oromia Region, Central Ethiopia. The ATH is the university hospital of the Arsi University and serves as a referral centre for a population of roughly 3.5 million in the Arsi and neighbouring zones. Hirsch Institute of Tropical Medicine (HITM) is a research and training centre of the Department of Gastroenterology, Hepatology and Infectious Diseases (DGHID) of Heinrich Heine University Düsseldorf, Germany, operated in cooperation with the Arsi University on the campus of ATH. All wards involved in perinatal and maternal care were included. These comprised gynaecology, obstetrics, paediatrics, and neonatology. The study was funded by the European ESTHER alliance (*Ensemble pour une Solidarité Thérapeutique Hospitalière en Réseau*) within a hospital partnership project to reduce perinatal and maternal morbidity and mortality due to infectious diseases.

### Study design

Ethics approval was obtained from College of Health Arsi University Ethical Review Committee (reference number A/U/H/C/120/10407/07). Additionally, written support from hospital leaders was obtained before starting project activities.

Activities consisted of three different phases in an uncontrolled before-and-after design.

Phase 1 is referred to as baseline assessment. It comprised a ward infrastructure survey, a HCWs’ perception survey, a hand hygiene knowledge questionnaire, and the observation of HCWs’ hand hygiene practices. The ward infrastructure survey was carried out involving senior members of the hospital management, the respective wards, and the study team. Within this survey, the availability of functional sinks was assessed and locations were identified where wall-fixed alcoholic handrub dispensers should be mounted. The HCWs’ perception survey, the hand hygiene knowledge questionnaire, and the observation form for hand hygiene practices were all designed by WHO [16]. Minor changes were made to the hand hygiene knowledge questionnaire to adapt to the local situation. English was the only language used in presentations as this is the language of medical education in Ethiopia. Question and answer sessions were held in English and Amharic. The observation of HCWs’ hand hygiene practices was carried out by two trained HCWs. Observations were performed only during day shifts for logistical reasons. The observers came to the wards at random times without prior announcement. They acted as unobtrusively as possible but disclosed their task readily on enquiry. Observation sessions lasted about 20 (±10) minutes. The purpose of breaking down the observation into sessions was to acquire an overview of practices [17]. Potential opportunities for hand hygiene according to the “my 5 moments of hand hygiene” were recorded and the actual number of episodes of hand hygiene. Hand washing referred to washing hands with either water alone or with soap and water, hand disinfection referred to the use of alcohol-based hand rub. Compliance with hand hygiene was calculated by the number of occasions when hand hygiene was performed divided by the number of total hand hygiene opportunities. All professional health care providers and students who were working in the selected wards were included in the study. HCWs were divided into two broad professional categories: (1) nurse/midwife/health officer/emergency surgeon (nurse with training in emergency surgery)/nurse student/midwife student/health officer student, (2) medical doctor/intern/medical student. The other two professional categories foreseen by WHO (auxiliary and other HCW) were not considered as they play a negligible role during patient care at ATH.

Phase 2 was the intervention. A four-day workshop was conducted with lectures on cultural aspects and scientific evidence of hand hygiene, and nosocomial infections in neonatology. Practical issues of the implementation of the multimodal hand hygiene improvement strategy were explained and baseline results of the HCWs’ perception survey and the hand hygiene knowledge questionnaire were presented. The workshop addressed hospital management, department heads, head nurses, focal persons for hygiene in the selected wards, and all interested staff of ATH. Four further half-day trainings were conducted to specifically address HCWs who could not attend the workshop. Additionally, all interns of ATH were explicitly invited to attend the trainings as they undergo rotations within the different wards and it was anticipated that some of them would be assigned to work on the wards where study activities were undertaken. HCWs who could not attend any training session were handed a pocket bottle with alcohol-based handrub after a short explanation of the concept “my five moments in hand hygiene”. Posters emphasizing the importance of hand hygiene were placed at strategic sites within ATH. WHO-recommended handrub formulations were produced according to WHO guidelines [8]. Demonstrations of the production were given by HITM staff during the workshop. Each HCW working in the pre-selected wards received a 100 ml pocket bottle filled with alcoholic handrub. A sticker on the pocket bottle indicated that once empty, refill for the bottle would be available at HITM. Wall-fixed hand disinfectant dispensers were mounted prior to the workshop and were filled immediately after the workshop with alcoholic handrub. Stickers on the containers indicated that refill would be available at HITM.

**Fig. 1:**
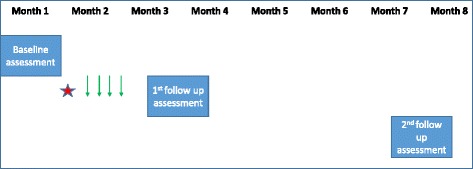
Time axis of study procedures. NOTE: Red star denotes workshop accompanied by placement of posters, filling of wall-fixed handrub dispensers, and distribution of pocket bottle hand disinfectants; thin green arrows denote half-day trainings for HCWs who could not attend the workshop. The HCWs’ perception survey was performed at baseline and at first follow up but not at second follow up

Phase 3 was the follow up assessment. The hand hygiene knowledge questionnaire was repeated immediately after the workshop or the training sessions, respectively. Starting from six weeks after the intervention, the HCWs’ perception survey and the observation of hand hygiene activities were repeated as described above. Study results were presented to hospital management and all concerned HCWs. An award was issued to the ward, which reached highest compliance with hand hygiene in the first follow up. Responsibility for the hand hygiene project was gradually transferred to ATH. A second follow up assessment was performed by only one observer (TN) starting from three months after the first follow up additionally recording the use of gloves but not recording the type of ward (Fig. 1).

### Statistical analysis

To detect a difference of 10% between rates of hand hygiene compliance before and after the implementation, 286 opportunities for hand hygiene had to be observed at baseline and follow up for each category [17]. Compliance at baseline and at follow up overall and for the different professional categories was compared with *χ* ^2^ tests. Multivariable logistic regression was used with the observation period (before or after the intervention) as the main independent variable. Type of ward and professional category were included as potential confounders. These confounders were chosen as in most studies it has been shown that compliance with hand hygiene varied by hospital ward and professional category, with higher compliance among nurses compared to doctors [18].

Hand hygiene knowledge questionnaire scores were calculated for each participant as the sum of all correct answers (each correct answer equalling 1 point). Results are expressed as medians and interquartile ranges (IQRs). Statistical significance was assessed by Wilcoxon rank-sum test, as participants were anonymous and pairing was therefore impossible. Results from the HCWs’ perception survey are shown as medians and IQRs of points given by participants on the 7-point Likert scale and were assessed by Wilcoxon rank-sum test. Data analysis was performed using EpiInfo, version 3.5.4 and SPSS, version 20. Two-tailed p values of less than 0.05 were considered to indicate statistical significance.

## Results

### Observation of compliance with hand hygiene

At baseline, first, and second follow up 146, 167, and 212 observation sessions were conducted, respectively. The median observation time per session was 22 (IQR 15–26) and 20 (IQR 16–25) minutes at baseline and first follow up, respectively. The duration of the observation sessions was not recorded during the second follow up.

### Education

A total of 164 HCWs participated in the trainings. All participants were listed and cross matched with registration lists. Due to intense staff rotation and unclear spelling of names we cannot give an exact number of how many HCWs were assigned to work in the respective wards during our study activities. We estimate, however, that at least 90% of the targeted HCWs were trained.

### Compliance with hand hygiene

A total of 7997 hand hygiene opportunities were assessed at baseline and follow up. Compliance with hand hygiene was 1.4% at baseline and rose to 11.7% and 13.1% in the first and second follow up, respectively. A significant increase in compliance with hand hygiene was seen across both professional categories and in all indications except for “before aseptic procedures” in the first follow up (Table 1). The increase in compliance with hand hygiene was associated with the intervention (crude odds ratio, 9.19: 95% confidence interval (CI) 6.62-12.77; p < 0.001). This association remained significant after adjustment for the potential confounders ‘type of ward’ and ‘professional category’ (adjusted odds ratio, 9.18; 95% CI 6.61-12.76; p < 0.001). Compliance with hand hygiene in the neonatology ward was higher compared to the paediatric ward as a reference at baseline (3.6% vs. 1.0%, p = 0.001) and at first follow up (24.5% vs. 6.2%, p < 0.001). Out of 41 hand hygiene actions at baseline 23 (56.1%) were handrub and 18 (43.9%) were hand washing, whereas at follow up handrub accounted for 619 (98.4%) and hand washing for 10 (1.6%) out of 629 hand hygiene actions, respectively.

**Table 1:** Compliance with hand hygiene at baseline and at follow up

Variable	Baseline			First follow up				Second follow up			
	No of HH actions	No of HH opportunities	Compliance (%)	No of HH actions	No of HH opportunities	Compliance (%)	*P* value^1^	No of HH actions	No of HH opportunities	Compliance (%)	*P* value^1^
Over all	41	2888	1.4	335	2865	11.7	<0.001	294	2244	13.1	<0.001
Professional category											
Category I	25	1470	1.7	157	1387	11.3	<0.001	73	678	10.8	<0.001
Category II	16	1418	1.1	178	1478	12.0	<0.001	221	1566	14.1	<0.001
Nurse	14	516	2.7	103	986	10.4	<0.001	63	461	13.7	<0.001
Midwife	6	284	2.1	46	324	14.2	<0.001	5	61	8.2	0.029
Medical doctor	9	285	3.2	27	275	9.8	0.002	70	306	22.9	<0.001
Intern	7	1109	0.6	151	1205	12.5	<0.001	151	1265	11.9	<0.001
Ward											
Paediatrics	9	882	1.0	50	807	6.2	<0.001	*ND*	*ND*		
Neonatology	24	672	3.6	166	677	24.5	<0.001	*ND*	*ND*		
Gynaecology	3	651	0.5	17	664	2.6	0.002	*ND*	*ND*		
Obstetrics	5	683	0.7	102	717	14.2	<0.001	*ND*	*ND*		
Indication											
b. p. c.	8	550	1.5	40	459	8.7	<0.001	10	86	11.6	<0.001
b. a. p.	2	275	0.7	5	305	1.6	0.455	20	422	4.7	0.003
a. b. f. e.	10	258	3.9	113	398	28.4	<0.001	5	10	50.0	<0.001
a. p. c.	15	1208	1.2	149	923	16.1	<0.001	130	924	14.1	<0.001
a. c. p. s.	6	597	1.0	28	780	3.6	0.002	129	804	16.0	<0.001

Only the major professional categories are displayed. Therefore, the sum of opportunities for these categories does not equal the total number of opportunities. Category I: nurse/midwife/health officer/emergency surgeon/nurse, midwife, health officer student; Category II: medical doctor/intern/medical student. NOTE: HH, hand hygiene; b.p.c., before patient contact; b.a.p., before aseptic procedure; a.b.f.e., after body fluid exposure; a.p.c., after patient contact; a.c.p.s., after contact with patient surroundings; ND, no data. ^1^Detemined by *χ* ^2^ tests with compliance at baseline as the reference.

The use of gloves was solely assessed during the second follow up. Gloves were used in 393 out of the 422 (93.1%) indications before aseptic procedures whereas gloves were used in 76 out of 1822 (4.2%) indications in the remaining four indications.

### Hand hygiene knowledge questionnaire

**Fig. 2:**
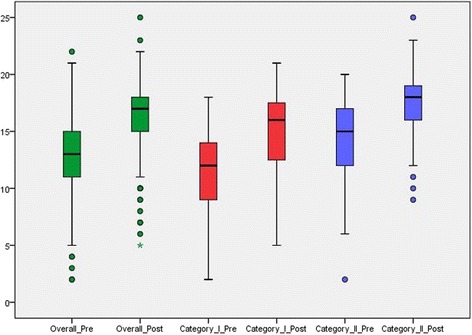
Knowledge of hand hygiene before and after the training. Box plot of overall scores (maximum score 25); 5%, 25%, 50%, 75%, 95% percentiles and outliers (circles); asterix denotes two outliers with equal scores. NOTE: Pre, before the intervention; post, after the intervention. Category I: nurse/midwife/health officer/emergency surgeon/nurse, midwife, health officer student; Category II: medical doctor/intern/medical student

The hand hygiene knowledge questionnaire was distributed before and immediately after the training sessions. A total of 141 HCWs filled the questionnaire before the training. Out of these, 70 HCWs belonged to category I and 61 HCWs belonged to category II. The remaining 10 participants did not belong to either of the predefined categories. After the training, the questionnaire was filled by a total of 139 HCWs (category I: 67; category II 63; neither category 9). The median knowledge score for all participants was 13 (IQR 11–15) at baseline and increased to 17 (IQR 15–18) after training (p < 0.001). Knowledge scores for category I and II at baseline were 12 (IQR 9–14) and 15 (IQR 12–17) and increased to 16 (IQR 12–18) (p < 0.001) and 18 (IQR 16–19) (p < 0.001), respectively (Fig. 2).

### Health-care workers’ perception of the strategy

At baseline and at first follow up, 100 HCWs’ perception surveys were handed to focal persons for hygiene for further distribution among HCWs. The return rates at baseline and follow up were 61% and 53%, respectively (Table 2).

**Table 2:** HCWs’ perception of the 5 components of the WHO multimodal hand hygiene improvement strategy

	Baseline	Follow up	*P* value^a^
No of respondents	61	53	
Category I	42	20	
Category II	19	33	
Strategy Component^b^			
*System change*:			
The healthcare facility makes alcohol-based handrub available at each point of care	6 (1–7)	6 (3–7)	0.359
*Education*:			
Clear and simple instructions for hand hygiene are made visible for every HCW	6 (3–7)	6 (4–7)	0.138
Each HCW is trained in hand hygiene	5 (3–7)	6 (3–7)	0.165
*Feedback*:			
HCWs regularly receive the results of their hand hygiene performance	4 (2–7)	5 (2–7)	0.969
*Workplace reminders*:			
Hand hygiene posters are displayed at point of care as reminders	3 (2–7)	6 (5–7)	<0.001
*Patient safety climate*:			
Leaders at your institution support and openly promote hand hygiene	5 (2–7)	6 (4–7)	0.062
Patients are invited to remind HCWs to perform hand hygiene	4 (1–7)	2 (1–7)	0.719

Category I: nurse/midwife/health officer/emergency surgeon/nurse, midwife, health officer student; Category II: medical doctor/intern/medical student. NOTE: HCW, health-care worker; WHO, World Health Organization. HCWs were asked to respond to the listed statements following the introductory question: “In your opinion, how effective would the following actions be to increase hand hygiene permanently in your institution?” ^a^Determined by Wilcoxon rank-sum test. ^b^Data show median scores (IQR) on a 7-point Likert scale (with extremes labelled as “not effective” at the lower and “very effective” at the higher end)

Before the training and at first follow up, the effectiveness of hand hygiene was judged to be high or very high by more than 90% of the HCWs. Self-assessment of compliance with hand hygiene revealed high estimates (70% compliance with hand hygiene at baseline and follow up) that did not match with observation results (see Additional file 1).

After the training, HCWs perceived the impact and different elements of the multimodal hand hygiene campaign to be very positive. All questions asked in the HCWs perception survey received median scores of 7 on the 7-point Likert scale, thus indicating maximum agreement (Table 3).

**Table 3:** HCWs’ perception about impact and different elements of the multimodal hand hygiene improvement strategy

	Median (IQR)
Has the use of alcohol-based handrub made hand hygiene easier to practice in your daily work?	7 (6–7)
Is the use of alcohol-based handrub well tolerated by your hands?	7 (6–7)
Did knowing the results of hand hygiene observation in your ward help you to improve your hand hygiene practices?	7 (5–7)
Has the fact of being observed made you paying more attention to your hand hygiene practices?	7 (4–7)
Were the educational activities that you participated in important to improve your hand hygiene practices?	7 (6–7)
Has your awareness of your role in preventing HAIs by improving your hand hygiene practices increased during the current hand hygiene promotional campaign?	7 (6–7)

NOTE: IQR, interquartile range; HAI, health-care associated infection. Results are shown as median scores on a 7-point Likert scale (with extremes labelled as “not at all” at the lower end and “very important” or “very much” at the higher end)

### Consumption of alcohol-based hand rub

**Fig. 3:**
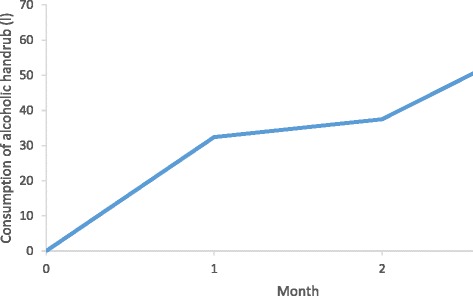
Consumption of alcohol-based handrub. Consumption of alcohol-based handrub in selected wards of Asella Teaching Hospital from the intervention (month 0) until the end of the first follow up. Display of total monthly consumption in litres

The consumption of locally produced alcohol-based handrub was solely recorded at HITM. It increased steadily after the training until the end of the first follow up (Fig. 3). Before the intervention, alcohol-based solutions were produced in the hospital pharmacy and responsibility for production of alcoholic handrub was transferred to ATH after the first follow up. Therefore, the consumption of alcohol-based disinfectants can only be indicated as shown.

## Discussion

In our study, we found a very low compliance with hand hygiene at baseline. Compliance at baseline was similar to two studies that had been undertaken in Ethiopia [19, 20] but was lower compared to a study from Bamako, Mali [21]. The main reason for the low baseline compliance appears obvious: hand hygiene products and facilities were not available on the wards. Alcoholic disinfectants were only used for disinfection of patients’ skin prior to aseptic procedures. For this purpose, usually one bottle of gentian violet-stained alcoholic solution was provided on each ward. The accessibility of soap and water was similarly difficult. The majority of sinks were non-functional for different reasons. Furthermore, the water supply of ATH was limited. This was especially true during the dry season when water supply was completely cut for several days in a row on various occasions.

In addition to the lack of alcoholic handrub and water – although presumably less important – compliance with hand hygiene at baseline may have been low for social reasons. One senior physician mentioned that he was reluctant to use his own pocket bottle hand disinfectant in order not to create envy and shame among other HCWs.

Observation at follow up showed a significant increase of compliance with hand hygiene. This increase was consistent across both predefined professional categories and in all four wards and persisted in the second follow up after responsibility for hand hygiene had been transferred to ATH. Improvement was associated with the intervention and this association remained significant after adjustment for potential confounders. However, compliance with hand hygiene remained low compared to data from developed countries. In a landmark study, which was conducted in the University of Geneva Hospitals, compliance with hand hygiene rose from 47.6% at baseline to 66.2% over a four-year period [3]. Particularly high rates of compliance with hand hygiene were observed in selected sites with vulnerable patients like intensive-care units after the implementation of a hand hygiene campaign [22, 23]. It must be considered, however, that in these studies only specific indications for hand hygiene were assessed (e.g. after completion of patient contact, on entrance into the unit). The concept “my five moments in hand hygiene” was not applied. One important study assessed the implementation of WHO’s hand hygiene improvement strategy in five countries with different socio-economic background [14]. Overall compliance before the intervention was significantly lower in low-income and middle-income countries than in high-income countries.

Compliance with hand hygiene improved across all indications except for “before aseptic procedures” in the first follow up. To our knowledge, this finding was not reported from previous studies. After having noticed preferential use of gloves before aseptic procedures at baseline and at first follow up, the use of gloves was systematically assessed during the second follow up. Observation revealed a high percentage of use of gloves (>90%) solely in the indication ‘before aseptic procedures’. The failure to change or remove contaminated gloves has been identified as major component of inadequate infection control practices [24]. We had emphasized the need for hand hygiene regardless of the use of gloves in our trainings. However, data show that this imperative was not understood and must be stressed further. Compliance with hand hygiene was highest after body fluid exposure and after patient contact. This possibly reflects the HCWs’ priority for self-protection rather than for protection of the patients. Self-protection has been shown to be the engine for hand hygiene adherence in several studies [5, 14, 25].

Interestingly, compliance with hand hygiene was similar in HCWs from category I and category II. This is in contrast to many studies, which found lower compliance in doctors than in nurses [5, 9], although Allegranzi et al. observed better compliance with hand hygiene in doctors than in nurses in Mali [21]. They hypothesized that the better compliance with hand hygiene in doctors could be due to a higher level of education and a stronger perception of their professional role [14]. The compliance with hand hygiene was higher in the neonatology ward when compared to the paediatrics ward as a reference. One possible reason for this – apart from the presumptions that hygiene is of critical importance in neonatology and many HCWs in neonatology may be particularly dedicated to their work - may have been the presence of a professor who emphasized a lot the importance of hand hygiene in routine patient care. It has been shown that role models may influence compliance with hand hygiene [26]. In our case the professor was a person that many HCWs looked up to and thus at least some HCWs may have tried to copy his behavior.

We produced alcohol-based handrub locally. Costs of local production were less than one fifth compared to commercially bought products. The skin tolerability of the handrub was perceived to be very good. After the intervention, hand hygiene actions were almost exclusively performed with handrub indicating high acceptance. The consumption of alcohol-based handrub increased steadily from the intervention until the end of the first follow up. The amount of alcohol-based handrub used was selected as indirect marker for compliance with hand hygiene in many settings although assessment based on product consumption cannot determine whether hand hygiene actions are performed in the right indications [5].

There were several challenges in our setting, which may have hampered achievement of better compliance with hand hygiene. First, we faced intense staff rotation on all levels within ATH. Since the beginning of the planning process until the end of the second follow up assessment, the position of medical director of ATH changed three times. Staff rotation on the wards resulted in observation of entirely different teams at baseline and follow up. Second, during the follow up assessment we were frequently shown empty wall-fixed alcoholic handrub dispensers and empty private pocket bottle hand disinfectants. Refill of alcoholic hand-rub had occasionally been fetched at HITM but then no further distribution among HCWs of the respective ward had been undertaken. On one ward, alcoholic handrub had been locked and was only accessible for one HCW. Third, although we did not measure the consumption of alcoholic handrub from wall-fixed dispensers and from pocket bottles independently, we felt that wall-fixed dispensers were used preferentially. According to our ward infrastructure, wall-fixed dispensers were mounted in selected sites with intense patient care. It seems obvious that the provision of wall-fixed dispensers to every room where patient care is performed would have been preferable. The concentration solely on the provision of alcohol-based handrub was regarded as a limitation by many HCWs and parts of the management of ATH. Whitby and McLaws demonstrated, however, that improved accessibility to sinks does not lead to improvement in compliance with hand hygiene [27]. In addition, WHO recommends the combined provision of pocket bottles and wall-fixed dispensers filled with alcohol-based handrub without focusing on water supply [5]. Just before the end of this study, management of ATH implemented a hygiene committee. The committee took over responsibilities like identification of further structural necessities. It has been shown that designated staff is one major critical component of an effective infection control program [28].

Results of the hand hygiene knowledge questionnaire were significantly better after the training than before the training. The improvement was seen in both professional categories and was similar to the improvement detected in a study from Bamako, Mali [21]. It may seem surprising that even immediately after the training the median scores reached were still far below the possible maximum score. We found that the way several questions should have been answered was not understood by many examinees (e.g. in some questions it is stated “tick one answer only” whereas in others there is no such statement. Many examinees wrongly only ticked one answer in these questions, too and therefore lost the chance to reach higher scores). We had already adapted the questionnaire to the local situation in accordance with WHO recommendations. However, some structural modifications may be necessary especially for HCWs who are not used to multiple choice exams and may be confused by the changing design of the questions. In further studies, current tools could be compared with adapted tools to confirm or refute our concern.

In contrast to Allegranzi et al., who detected significant increases in median perception scores for all five components of the multimodal WHO hand hygiene improvement strategy, we only found increased median scores in the component “reminders at the workplace” [21]. This may be explained by the high baseline scores in our study. HCWs estimated their compliance with hand hygiene and the compliance of their co-workers to be high. Estimates differed greatly from our observation findings. This is in line with various studies, which reported that the correlation between self-assessment and observation findings is low [29, 30]. The acceptance of the different elements of the hand hygiene campaign and the perceived impact of hand hygiene were very high as indicated by maximum scores after the training. This finding supports the multimodal approach recommended by WHO. We cannot know, however, which element was most important for the observed outcomes and how the outcomes would have been if one or several elements had been omitted.

Our study has several limitations. First, although English is the official language of medical education in Ethiopia, not all HCWs have good English language skills. HCWs insisted on English presentations and WHO could not provide working materials in Amharic. We managed to establish question and answer sessions in Amharic after each training but we cannot exclude that outcomes would have been better if Amharic had been used preferentially.

Second, it seems logical that HCWs are reminded of performing hand hygiene actions in the presence of an observer. Observation at second follow up was entirely performed by local staff (TN), whereas international staff (NS) did most of the observation at baseline and at first follow up. Assessments of different observers may vary. However, all observers were well trained in WHO hand hygiene observation methods, and criteria defined by WHO are straightforward to minimize inter-observer differences [17].

Third, the hospital management addressed the wish of extension of our hygiene activities to the entire hospital arguing that all HCWs and patients should benefit from the positive effects of proper hand hygiene. We felt that our approach was adequate as pilot project to demonstrate feasibility and efficacy. Hospital management was in charge of ensuring sustainability of the project and of extending activities to the ward that had not yet been covered.

Fourth, we performed two follow up assessments in relatively short time intervals after the intervention. It would have been preferable to perform a time series analysis with several follow ups to longitudinally assess compliance with hand hygiene.

Last but not least, the clinical relevance of our intervention remains unclear in the light of compliance rates that were still low at follow up. To assess the rates of HAIs in ATH surveillance activities would have to be implemented. Surveillance is essential to record the burden of infectious diseases and the effect of interventions. Moreover, by itself, surveillance can lead to reduction in HAIs [31]. The most widely used surveillance definitions for HAIs come from the Centers for Disease Control and Prevention (CDC) and the National Healthcare Safety Network [32]. They are rarely applied in low-income countries as strict criteria have to be used including bacterial culture in most settings. Future research may help to develop criteria, which are adapted to the settings in resource-constrained countries. Ideally, prospective investigations should assess both compliance with hand hygiene and rates of HAIs.

## Conclusion

We successfully implemented the WHO multimodal hand hygiene improvement strategy in selected wards of ATH. The intervention was highly appreciated by participating HCWs. The increase in compliance with hand hygiene persisted after responsibility for the project had been transferred to ATH. A time series analysis should be performed to further assess the longitudinal evolution of compliance with hand hygiene. Compliance with hand hygiene was low compared to similar projects. Simultaneous surveillance of HAIs could help to assess the clinical impact of such interventions.

## Additional files

Additional file 1: HCWs perception. (XLSX 13 kb)
Additional file 2: Observation raw data total numbers. (SAV 806 kb)
Additional file 3: Hand hygiene knowledge questionnaire pre and post. (SAV 4 kb)
Additional file 4: Perception survey. (SAV 54 kb)
Additional file 5: Alcoholic handrub. (XLSX 10 kb)

## Abbreviations

ATH: Asella Teaching Hospital
CDC: Centers for Disease Control and Prevention
CI: Confidence interval
DGHID: Department of Gastroenterology, Hepatology and Infectious Diseases
ESTHER: Ensemble pour une Solidarité Thérapeutique Hospitalière en Réseau
HAI: Health-care associated infection
HCW: Health-care worker
HITM: Hirsch Institute of Tropical Medicine
IQR: Interquartile range
WHO: World Health Organization
